# Draft Genome Sequence of Plant Growth-Promoting Burkholderia sp. Strain BE12, Isolated from the Rhizosphere of Maize

**DOI:** 10.1128/genomeA.00299-18

**Published:** 2018-04-26

**Authors:** Qassim Esmaeel, Lisa Sanchez, Mathilde Robineau, Stephan Dorey, Christophe Clément, Cédric Jacquard, Essaid Ait Barka

**Affiliations:** a Unité de Résistance Induite et Bioprotection des Plantes–EA 4707, UFR Sciences Exactes et Naturelles, University of Reims-Champagne-Ardenne, Reims, France

## Abstract

Burkholderia sp. strain BE12, isolated from a French agricultural soil, possesses antifungal activity against a set of phytopathogenic fungi and has friendly interactions with grapevine. Here, we present the draft genome sequence of BE12, along with genes related to plant growth-promoting traits and siderophores that this strain contains, supporting its plant growth and antifungal activities.

## GENOME ANNOUNCEMENT

The genus Burkholderia comprises more than 90 species that are distributed in a wide range of environments, including water, soil, plants, animals, and humans (1–4). The capacity of Burkholderia spp. to live in different ecological niches is due to their large genome size, ranging from 3 to 9 Mb, divided into 3 chromosomes and up to 5 plasmids (5). Several species of Burkholderia are well known for their potential role in plant growth promotion and protection in plants against soilborne pathogens (6–8). Thus, the capabilities of Burkholderia species to reduce yield losses and promote growth have led to increased interest in the use of Burkholderia strains as biocontrol and biostimulant microorganisms in agriculture. The strain described here, Burkholderia sp. BE12, was isolated from a French agricultural soil. In dual confrontation assays, this bacterium showed an inhibitory effect against Botrytis cinerea, Fusarium oxysporum, and Rhizoctonia solani.

This strain also has been screened and characterized, *in vitro*, for its potential plant growth-promoting (PGP) traits, including siderophore production, phosphate solubilization, and the production of phytohormones. An *in vivo* assay showed that Burkholderia sp. BE12 caused a significant increase in growth parameters of grapevine (Vitis vinifera L.) and was able to reduce the development of gray mold, caused by B. cinerea, on grapevine plantlets (unpublished data).

To better understand the PGP and biocontrol effects of this strain, the whole genome was sequenced. Total DNA was extracted using the Wizard genomic purification DNA kit (Promega Corp., Madison, WI, USA) and sequenced at MicrobesNG (http://www.microbesng.uk) using Illumina MiSeq and HiSeq 2500 technology platforms, with 2 to 250-bp paired-end reads. The closest existing reference genome was determined using Kraken (9), and the reads were mapped using the Burrows-Wheeler Aligner (BWA) MEM algorithm (http://bio-bwa.sourceforge.net) to assess data quality. The reads were assembled by *de novo* assembly using SPAdes (http://cab.spbu.ru/software/spades/). The draft genome of 7,472,757 bp includes 97 contigs with a GC content of 66.94% and an *N*_50_ contig size of 180,386 bp. Gene function prediction was performed by the Rapid Annotations using Subsystems Technology (RAST) server (http://rast.nmpdr.org) (10) followed by an annotation using the SEED database (11), resulting in 71 RNAs and 7,040 coding sequences.

*In silico* analysis using antiSMASH (12) and RAST revealed the presence of a siderophore gene cluster and genes involved with the production of indole acetic acid (IAA), a plant hormone associated with plant growth (13). Furthermore, the genome sequence indicates the presence of pyrroloquinoline quinone synthase and glucose dehydrogenase (implicated in the production of gluconic acid) and 2-ketogluconic acid production (involved in mineral phosphate solubilization) (14). The annotated genome also has 15 genes related to *N*-acylhomoserine lactone, which is involved in the quorum-sensing system, and 1 gene related to 1-aminocyclopropane-1-carboxylate (ACC) deaminase, which potentially plays a role in promoting plant growth (15). In addition, the genome has genes contributing to cell wall degradation, such as endoglucanase and cellulase, and motility proteins. All these features may explain the ability of Burkholderia sp. BE12 to successfully promote plant growth and protect plants from disease.

### Accession number(s).

This whole-genome shotgun project has been deposited in GenBank under the accession no. PREY00000000. The version described in this paper is the first version, PREY01000000.
